# Host niche may determine disease-driven extinction risk

**DOI:** 10.1371/journal.pone.0181051

**Published:** 2017-07-13

**Authors:** Mark Blooi, Alexandra E. Laking, An Martel, Freddy Haesebrouck, Merlijn Jocque, Tom Brown, Stephen Green, Miguel Vences, Molly C. Bletz, Frank Pasmans

**Affiliations:** 1 Department of Pathology, Bacteriology and Avian Diseases, Faculty of Veterinary Medicine, Ghent University, Merelbeke, Belgium; 2 Operation Wallacea, Hope House, Old Bolingbroke, Lincolnshire, United Kingdom; 3 Hopkins Marine Station, Stanford University, Pacific Grove, California, United States of America; 4 Centre for Applied Zoology, Cornwall College Newquay, Cornwall, United Kingdom; 5 Zoological Institute, Technische Universität Braunschweig, Braunschweig, Germany; Vanderbilt University School of Medicine, UNITED STATES

## Abstract

The fungal pathogen *Batrachochytrium dendrobatidis* (*Bd*) drives declines and extinctions in amphibian communities. However, not all regions and species are equally affected. Here, we show that association with amphibian aquatic habitat types (bromeliad phytotelmata versus stream) across Central America results in the odds of being threatened by *Bd* being five times higher in stream microhabitats. This differential threat of *Bd* was supported in our study by a significantly lower prevalence of *Bd* in bromeliad-associated amphibian species compared to riparian species in Honduran cloud forests. Evidence that the bromeliad environment is less favorable for *Bd* transmission is exemplified by significantly less suitable physicochemical conditions and higher abundance of *Bd*-ingesting micro-eukaryotes present in bromeliad water. These factors may inhibit aquatic *Bd* zoospore survival and the development of an environmental reservoir of the pathogen. Bromeliad phytotelmata thus may act as environmental refuges from *Bd*, which contribute to protecting associated amphibian communities against chytridiomycosis-driven amphibian declines that threaten the nearby riparian communities.

## Introduction

Chytridiomycosis, caused by the chytridiomycete fungus, *Batrachochytrium dendrobatidis* (*Bd*) [1], drives global amphibian declines and extinctions [2–5] and is currently considered the greatest infectious disease threat to biodiversity [6,7]. Although *Bd* has a worldwide distribution with confirmed presence in over 500 amphibian species from 52 countries [6,8], its most devastating impact is centered on specific regions, such as mountainous regions in Central America [3,9]. In these areas, the cumulative time individuals of a species spend in riparian habitats significantly increases the likelihood of decline [5,9–12]. Therefore, even in these hotspots of chytridiomycosis-driven declines, a proportion of species are less affected [9,13,14], especially those with arboreal, bromeliad-associated habits [15–18]. The reasons for these differential infection and disease dynamics of *Bd* have been suggested to be multifactorial and dependent on local environmental-, host- and pathogen-associated factors [13,19–24]. Since metamorph mortality disproportionately contributes to chytridiomycosis-driven declines [25], the habitat of juvenile stages and all associated mechanisms affecting *Bd* presence, survival and transmission in these habitats is expected to play a major role in *Bd*-related amphibian declines. Mitchell et al. (2008) [26] predict that the longer the fungus persists in the aquatic environment, the greater its impact on host populations. Besides abiotic factors such as temperature and pH [22,24,27], the abundance and diversity of aquatic, *Bd*-ingesting microorganisms (predatory micro-eukaryotes) have recently been shown to dictate the *Bd* infection and disease dynamics in a European hotspot of chytridiomycosis by driving aquatic pathogen loads [28].

Here we test the hypothesis that the bromeliad environment provides a “safe haven” for amphibians from chytridiomycosis in comparison with the riparian habitats in Central American cloud forests by 1) comparing to what extent Neotropical bromeliad- versus stream-associated amphibians are threatened in their survival due to *Bd*. Additionally, we 2) assessed *Bd* infection levels of bromeliad- versus stream-associated amphibians in Cusuco National Park, Honduras and 3) evaluated which environmental parameters in streams and tank bromeliads help explain observed differences in *Bd* infection on these amphibians.

## Methods

### Correlation between conservation status of Central American anuran species and their association with bromeliads or streams

To compare the threat of chytridiomycosis in Central American bromeliad- and stream-associated anurans, a list was compiled based on amphibian species known to occur in this region [29–31] and based on known habitat usage. Only species known to inhabit or reproduce in bromeliad or stream environments were included (S1 Table). Species with reports of occasional or rare sightings in one of the two microhabitats were excluded. A distinction was made between association to a microhabitat and restricted habitat for larval development, for further comparison. Species that reproduce in streams as well as other waterbodies were included in the larval development category. The IUCN Red List of Threatened Species was utilized to determine the IUCN conservation category of each species, as well as the species major threats (S1 Table). IUCN categories were grouped: data deficient (DD), least concern and near threatened (LC & NT), threatened and vulnerable (EN & VU), and critically endangered or extinct (CR & EX). Threats were grouped into five categories: no threats, habitat loss, pet trade, pollution and *Bd*. Any mention of one of these threats for an anuran species whether it be future, suspected or proven, resulted in inclusion of the species into each corresponding category.

### Study site

This study was conducted in Cusuco National Park (CNP), Honduras. This park is approximately 234km^2^ in area, with elevation ranging from 500 m to 2425 m above sea level. CNP is located in north-western Honduras and is part of the Merendón mountain range. It is included in the Meso-American biodiversity hotspot and is characterized by high species richness [32] and ranked within the top 25 protected areas globally, of highest importance for conserving irreplaceable amphibian diversity [33]. CNP was chosen as the study site because of its large number of endemic amphibian species [34], of which several are IUCN red listed and negatively affected by *Bd* [18,25,32]. Widespread presence of *Bd* has been confirmed here in multiple species, but *Bd*-induced declines have been observed only for riparian amphibian species [18,25]. Sample collection took place between June and August in 2014 and 2015. Permission for the collection and export of all mentioned samples was obtained by Operation Wallacea (Hope House, Old Bolingbroke, Lincolnshire, UK) and the OpWall Honduran counterpart Expediciones y Servicios Ambientales de Cusuco (ESAC) and issued by the Secretaría de Estado en el Desoacho de Agricultura y Granadería (Number: 21839).

### Sample collection

To determine the prevalence of *Bd* in different aquatic environments and in amphibians, filtered water and amphibian skin or mouthpart swabs, depending on life stage, were collected from bromeliads and streams and their associated anurans around CNP’s base camp. To determine the ability of *Bd* to persist in different aquatic habitats, water samples from the same habitats were collected. The locations were chosen based on accessibility, presence of the desired microhabitat and comparable elevation levels.

#### Bromeliad and stream microhabitats

Thirty-two bromeliads and twelve sampling locations derived from the main stream running through CNP were selected for sampling. Large bromeliads (circumference >45 cm) were sampled in order to allow sufficient water volumes (30 ml) to be withdrawn and due to the preference of bromeliad-associated amphibians for larger specimens [16]. For each bromeliad and stream site, GPS coordinates, canopy openness (improved Moosehorn design [35]), water pH (pH test kit, JBL GmbH & co, Neuhofen Germany), water temperature and ambient air temperature were recorded. If animals were not present, water parameters were still taken, and if bromeliads spilled water the amphibians within were sampled, leading to a dataset with missing data for some of the respective parameters. While full data sets for all sampling sites were not available, the spatial distribution of our sampling is extensive enough to exclude random site effects. *Bd* prevalence in the bromeliads (n = 24) and streams (n = 5) was determined by filtering (0.45 μm syringe filters) 30 ml or 1 liter of water respectively, with subsequent storage of the filters in 70% ethanol until further processing (see below for sample processing). These filters were also used for micro-eukaryote diversity assessment using 18S amplicon-based next generation sequence analysis. Microscopic determination and abundance estimates of aquatic micro-eukaryotes were carried out on 1 ml of the collected water samples.

#### Amphibian samples

Prevalence of *Bd* in encountered amphibians was determined from non-invasively collected skin swabs in post-metamorphic individuals. For tadpoles, mouthparts were swabbed. For the swabbing technique established protocols were used [36,37]. Swabs were stored in 70% ethanol in order to preserve the samples in humid field conditions [25].

### Sample processing

#### Prevalence of *Batrachochytrium dendrobatidis*

DNA was extracted from swabs using 100 μl of Prepman Ultra DNA extraction buffer [36,38]. Ethanol was removed from the swabs prior to DNA extraction by centrifugation, disposal of the supernatant and leaving the samples open until evaporation of all ethanol had occurred in a laminar flow hood. DNA extraction from the water filters was carried out using the MoBio Powersoil^®^ DNA isolation kit (MoBio Laboratories, Carlsbad, USA) following the protocol of Walker et al. [39] with minor modifications as follows: As disposable syringe filters were used, filter housings were opened and the ethanol on the filters was allowed to evaporate prior to DNA extraction and the manufacturer’s protocol was followed for homogenization of samples (vortexing the samples instead of using a Mini-Beadbeater). All extracted DNA samples (derived from swabs and filters) were diluted 1/10 in HPLC water for reducing PCR inhibition [36] and stored at –20°C until further processing. Samples were processed using the *Bd*-specific real-time PCR described by Boyle et al. (2004) on a CFX96 real-time system (Bio-Rad Laboratories, Hercules, CA). To check whether PCR inhibition did occur, all DNA samples were assayed in a *Bd* PCR inhibition assay in which an internal positive control (IPC) was added to a single well for each sample [36]. If inhibition was detected in a sample, BSA was added to the real-time PCR mixture, and if this did not alleviate inhibition, samples were further diluted ten-fold until no inhibition could be detected [40]. Real-time PCR results (genomic equivalents (GE) of *Bd* zoospores as determined by comparing real-time PCR values to values obtained by assaying a standard dilution series of reference *Bd* DNA (JEL 423) were corrected for the applied dilution factor and converted to loads per swab or per filter.

#### Persistence of *Bd* in aquatic microhabitats

We compared the capacity of the 15 bromeliad and 15 stream water samples to reduce the number of viable *Bd* zoospores using previously described techniques [28,41]. Briefly, suspensions of viable and dead *Bd* zoospores (strain JEL423, 10^4^ zoospores/ml) were prepared. Death of heat-treated (85°C, 15 minutes) zoospores was confirmed by checking for growth over 10 days. *Bd* zoospore viability was assessed using the *Bd* ethidium monoazide (EMA) real-time PCR [42]. The conditions assessed were microhabitat (bromeliad, stream), filtration to selectively remove micro-eukaryotes over 5 μm in size (unfiltered, filtered (5 μm syringe filter, GE Healthcare Europe, Belgium)) and time point (0 hours, 24 hours). Controls composed of viable and dead *Bd* zoospores in distilled water were included. In short, 100 μl of *Bd* zoospore suspension was added to 1 ml of the sample conditions (microhabitat, filtration) in 48-well plates and incubated at 20°C. After 24 hours of incubation, 7.5 μl of EMA was added to 150 μl aliquots of all samples and controls and 142.5 μl of TGhL broth (final EMA concentration 25 μg/ml), and samples were incubated in the dark and subsequently photo activated (5 minutes, 500 Watt halogen light). EMA exposed samples were transferred to 1.5 ml tubes and washed twice in HPLC water by centrifugation (5000 rpm, 5 minutes, 20°C). After washing, DNA extraction of the pellet was carried out by resuspending it in 100 μl Prepman ultra and incubating at 100°C for 10 minutes. All samples were diluted 1/10 and stored at -20°C until further processing. All conditions were tested in duplicate, and processing of the samples occurred within 72 hours after collection. DNA extracts were processed using the *Bd*-specific real-time PCR [38] as specified in the previous section (including checking for the occurrence of PCR inhibition). Resulting values were used as an indication of *Bd* persistence, as only DNA from viable *Bd* cells is amplified with the EMA real-time PCR [41].

#### Micro-eukaryote diversity and abundance estimates using microscopy

To assess the abundance and community structure of micro-eukaryotes, 1 ml of water from each sampled microhabitat was visually examined drop by drop using a field microscope (Bresser microset). In the case of high numbers or fast movement of micro-eukaryotes one drop of Protoslo^®^ Quieting Solution (Carolina Biological Supply Company, Burlington, USA) was applied to the microscopy slide in order to improve taxonomic determination and abundance estimation. Determination of species was carried out using standard determination keys [43,44] and performed as close to species level as possible.

#### Micro-eukaryote diversity estimate using 18S rRNA amplicon-based sequencing

DNA extraction from the water filters was carried out as described earlier. A portion of the V9 region of the eukaryotic 18S rRNA gene was sequenced by adapting a dual-index approach [45]. Illumina adaptors and unique barcodes were included on both the forward (Euk_1391f) and reverse (Euk_B) primers [46,47]. PCR conditions and the amplification protocol followed the Earth Microbiome Project 18S protocol (http://www.earthmicrobiome.org/emp-standard-protocols/18S/, version 4_13). In short, PCR reactions contained 13 μl of DNA-free water, 10 μl 5 Prime MasterMix, 0.5 μl of each primer (10 μM), 4 μl of a blocking primer, and 1.0 μl of DNA. The blocking primer originally designed for mammals (see Earth Microbiome website) is effective for vertebrates in general and was used to minimize amplification of vertebrate genomic DNA. PCR conditions consisted of a denaturation step of 94°C for 3 minutes, followed by 35 cycles at 94°C for 45 seconds, 65°C for 15 seconds, 57°C for 30 seconds and 72°C for 90 seconds, and a final extension at 72°C for 10 minutes. All samples were then pooled together in approximately equal concentration (as determined by gel band strength), and purified with the Qiagen MiniElute Gel Extraction Kit. The DNA concentration was determined on a Qubit fluorometer using a broadrange dsDNA kit. Paired-end 2x250 v2 chemistry was used to sequence the samples on an Illumina MiSeq (Helmholtz Center for Infection Research, Braunschweig Germany).

All sequence processing was conducted using Quantitative Insights Into Microbial Ecology (MacQIIME v1.9.1, [48]). Forward and reverse reads from each sample were joined and quality filtered to remove low-quality sequences with the QIIME default quality parameters. Remaining good-quality sequences (471,056 sequences) were clustered into operational taxonomic units (OTUs) at 97% similarity using the UCLUST algorithm under an open reference OTU-picking strategy http://qiime.org/tutorials/open_reference_illumina_processing.html [49,50] The SILVA 119 release was used as the reference database for reference-based OTU clustering, aligning sequences and assigning taxonomy. The most abundant sequence from each OTU was selected as a representative sequence and these representative sequences were aligned using PyNAST [51]. Taxonomy was assigned using BLAST within QIIME. Samples were rarefied to 1000 reads per sample, allowing the majority of samples to be included in analyses. The OTU table was subsequently filtered in two ways: (1) to include only OTUs of small metazoans, including small arthropods and rotifers, and (2) to include only OTUs of protists.

#### Ingestion of *Bd* zoospores

We assessed to what extent the different organisms present in the water samples were capable of consuming *Bd* zoospores. A *Bd* zoospore suspension was prepared as described previously. CellTracker^™^ Green CMFDA (Life Technologies, Carlsbad, USA) was added to 1 ml of zoospore suspension (final concentration of the CellTracker 6μM). This mixture was then shielded from light from this step forward and incubated for 30 minutes at 20°C. The mixture was washed in 1 ml of distilled water by centrifugation (3000 rpm, 5 minutes) and incubated for 30 minutes at 20°C. In order to remove excess fluorescent marker, two additional washing steps composed of centrifugation (3000 rpm, 5 minutes) and resuspension in distilled water were performed. Affirmation of viability and fluorescent labelling of zoospores was done using an epifluorescence microscope. Ostracods (*Cypridopsis* spp.), copepods (*Cyclops* spp.), brown planaria (*Dugesia dorotocephala*), nematodes (*Caenorhabditis elegans*) and tardigrades (*Milnesium tardigradum*) were obtained from Carolina Biological Supply Company (Burlington, USA) and maintained as recommended by the supplier. These organisms were chosen due to their similarity to organisms found within the bromeliad and stream microhabitats. One ml of labelled zoospores was added to 1 ml of each of the 5 species of micro-eukaryotes and incubated for 2 hours at 20°C (final concentrations of 100 zoospores per 1 micro-eukaryote). A control prepared by adding fluorescent marker to distilled water (including all incubation and washing steps) was included to check for labelling of cells other than *Bd* zoospores. This control was in turn added to all five species of micro-eukaryotes and incubated for 2 hours at 20°C. Another control was included by adding unlabeled zoospores to the 5 micro-eukaryote species. All combinations were assessed in triplicate. Using an epifluorescence microscope, sample slides were visually screened for ingestion of labelled *Bd* zoospores, without quantification. Fluorescence imaging does not allow the ingested zoospores to be quantified due to the large numbers of (aggregated) zoospores inside the micro-eukaryotes. However, when ingestion took place, it was common throughout the organisms in the sample.

### Statistical analysis

Odds Ratios (ORs), and corresponding 95% confidence intervals, were calculated for the threat of *Bd*, as well as for the number of species in the “endangered” category, compared between bromeliad- and stream-associated species (S1 Table). To test for differences in *Bd* infection intensity, microeukaryote abundance and microeukaryote diversity between microhabitats, pairwise Mann Whitney Wilcoxon tests were performed. To compare prevalence of *Bd* between microhabitats a Fisher’s exact test was performed. For the amplicon-based 18S sequencing data, Chao1 diversity, Shannon diversity and Observed OTU richness were calculated for all samples in each of the OTU tables. Kruskal-Wallis tests were used to compare alpha diversity values between streams and bromeliads. To test for differences in environmental pH, *Bd* persistence, temperature and canopy openness scores between bromeliad and stream microhabitats, Mann—Whitney—Wilcoxon tests were used (all statistical analysis were performed in R, version 3.2.3) [52].

## Results

### Central American bromeliad-associated anuran species are predicted to be less threatened by *Bd* in comparison to their stream-associated counterparts

**Fig 1:**
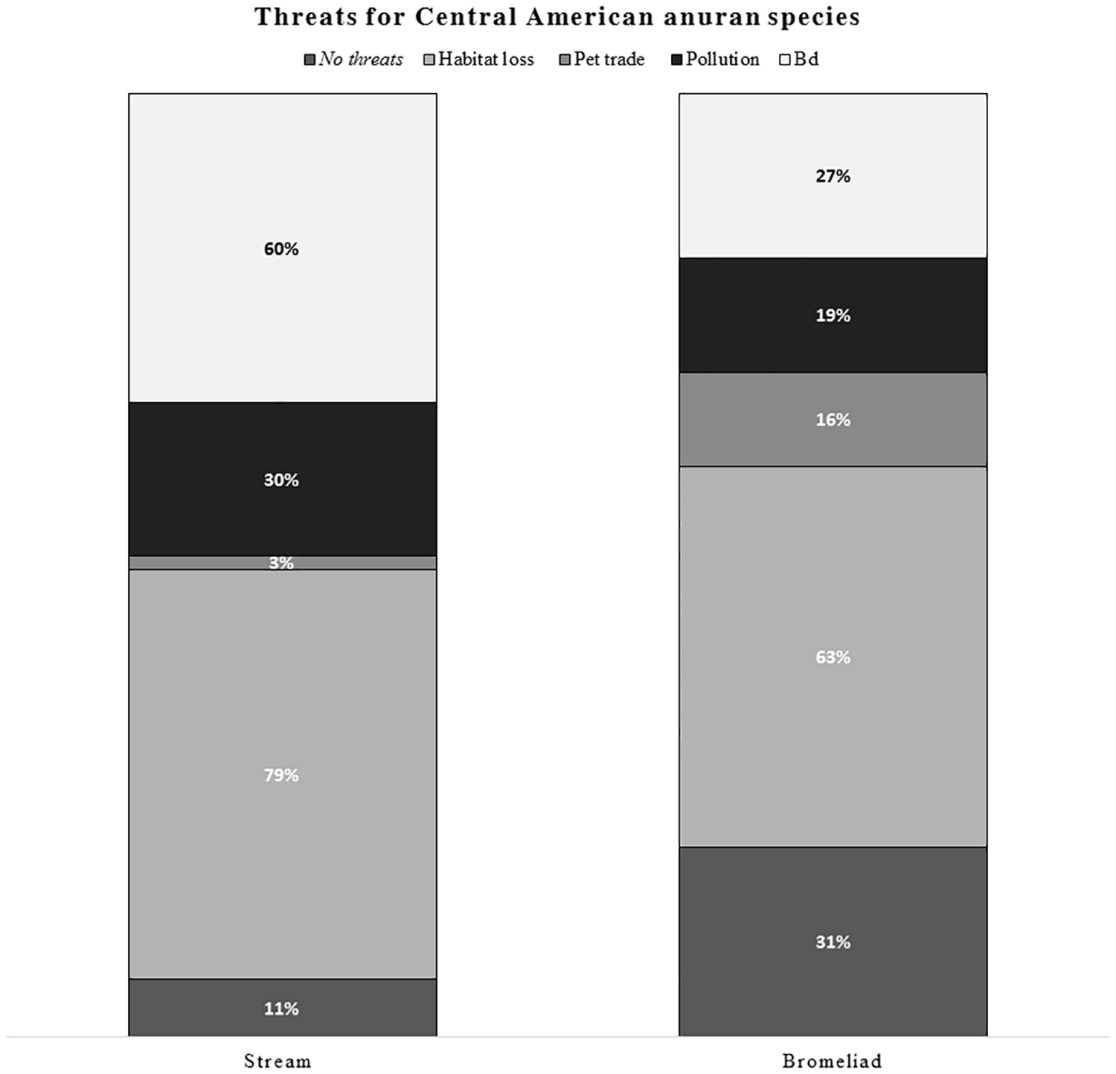
Threats for Central American bromeliad- and stream-associated anuran species. Bars indicate the total percentage of each of the five defined threats present for Central American anuran species within bromeliad and stream habitats. The chart is compiled from IUCN red list data (Also see S1 Table) (different threats are from bottom to top: no threats, habitat loss, pet trade, pollution and *Bd*). In this figure, *Plectohyla dasypus* is included among stream-dwelling species because it larvae develop in streams. However, adults of this species are often associated with bromeliads.

A comparison of all (184) Central American anurans showed higher numbers of species being threatened by *Bd* in streams compared to anuran species in bromeliads (60% and 27%, respectively) (Fig 1, S1 Table) based on IUCN data for major threats. A higher percentage of stream-associated anuran species is indexed in the critically endangered conservational category when compared to bromeliad-associated species (36% and 6%, respectively) (S1 Table). The odds of being threatened by *Bd* was nearly four times higher (OR: 3.71, 95% CI: 1.5985–8.5985) in stream-associated anurans than in bromeliad-associated anurans. The odds of being in the critically endangered (CR + EX) IUCN conservational category is nearly 9 times higher (OR: 8.88, 95% CI: 2.05–38.54) in stream-associated anurans compared to bromeliad-associated anurans.

### Bromeliad microhabitats are less conducive for *Bd* infection in comparison to stream microhabitats

**Fig 2:**
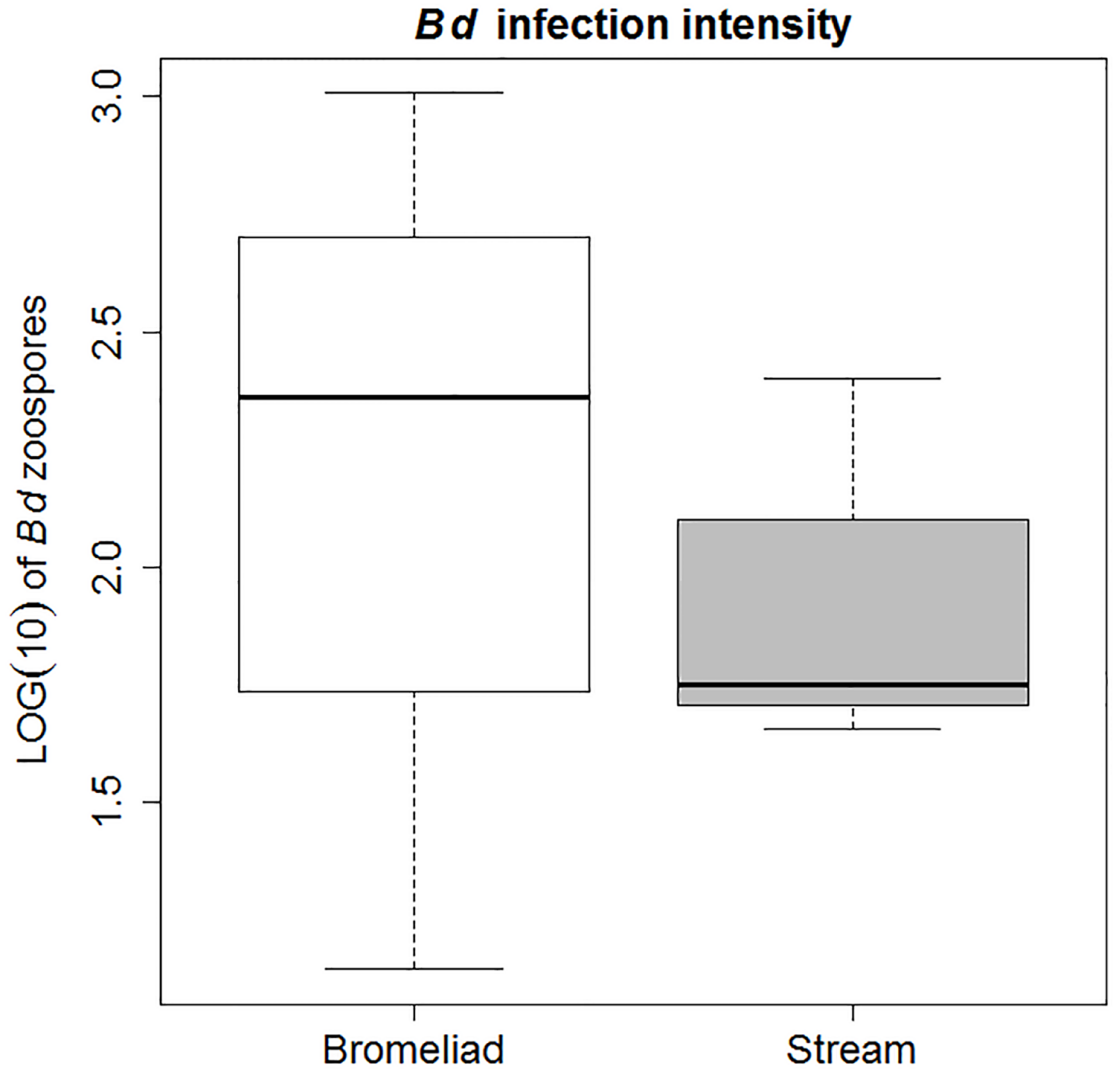
Boxplot showing infection intensity of *B*. *dendrobatidis* in the sampled amphibians. Median (bars), interquartile range (boxes) and total range (whiskers) are shown (Bromeliad n = 116, Stream n = 150).

From the total number of 24 stream sites and 41 bromeliad sites, we found and sampled a total of 116 amphibian individuals from 20 bromeliad sites, and 150 individuals from 18 stream sites. Prevalence of *Bd* in the sampled amphibians, ignoring site and species differences due to sparse data, was significantly lower in bromeliads (3.4%) in comparison to streams (12.4%) (Table 1, Fisher’s exact test, *p* = 0.008), while infection intensity did not differ significantly between the two microhabitats (Fig 2, W = 32, *p* = 0.23). While PCR inhibition did occur in the swab samples (11 *Bromeliohyla bromeliacia*, 9 *Ptychohyla hypomykter*, 3 *Cryptotriton nasalis* and 2 *Plectrohyla dasypus*), this could be alleviated in most samples by adding bovine serum albumin (BSA) to 1/10 diluted samples in the real-time PCR mixture. Samples that still showed inhibition after adding BSA (3 *B*. *bromeliacia*, 3 *C*. *nasalis*, 2 *P*. *hypomykter*, 1 *P*. *dasypus)* were further diluted in HPLC water to 1/100 dilutions, which removed PCR inhibition in all but one sample (*P*. *dasypus*) in which PCR inhibition was only alleviated after diluting the sample to 1/1000. No additional positive samples were obtained after alleviating PCR inhibition. None of the water samples tested positive for *Bd*. PCR inhibition occurred in 10 of the filtered-water samples (8 derived from bromeliads and 2 from streams), but adding BSA to the real-time PCR mixture alleviated this in all but one sample (bromeliad), which needed to be diluted to 1/1000 to remove PCR inhibition. Again, no additional positive samples were obtained after alleviating PCR inhibition.

**Table 1:** Prevalence of *B*. *dendrobatidis* in the sampled amphibians. Prevalence data of *B*. *dendrobatidis* in the sampled amphibians divided in amphibian taxa, life stages and species.

	Bromeliad					Stream				
		No. sampled	*Bd* positive	Mean infection intensity (Min -Max)	Prevalence (%)		No. sampled	*Bd* positive	Mean infection intensity (Min -Max)	Prevalence (%)
**Amphibian taxa**	Anura	106	4	372 (14–1014)	3.7	Anura	150	19	101 (28–252)	12.7
	Caudata	10	0	0	0	Caudata	0	0	0	0
	Total	116	4	372 (14–1014)	3.4	Total	150	19	101 (28–252)	12.7
**Life stages**	Larvae	94	4	372 (14–1014)	4.3	Larvae	135	14	117 (48.2–252)	10.4
	Metamorphs	2	0	0	0	Metamorphs	11	5	71 (28.1–105.2)	45.4
	Juveniles	4	0	0	0	Juveniles	3	0	0	0
	Adults	16	0	0	0	Adults	1	0	0	0
**Species**	*Bromeliohyla bromeliacia*	106	4	372 (14–1014)	3.8	*Plectrohyla exquisita*	3	2	233 (218–248)	66.7
	*Cryptotriton nasalis*	10	0	0	0	*Plectrohyla dasypus*	45	7	107.2 (49.8–252)	15.6
						*Ptychohyla hypomykter*	82	9	72.4 (28.1–204)	11
						*Duellmanohyla soralia*	21	1	49.8	4.8

**Fig 3:**
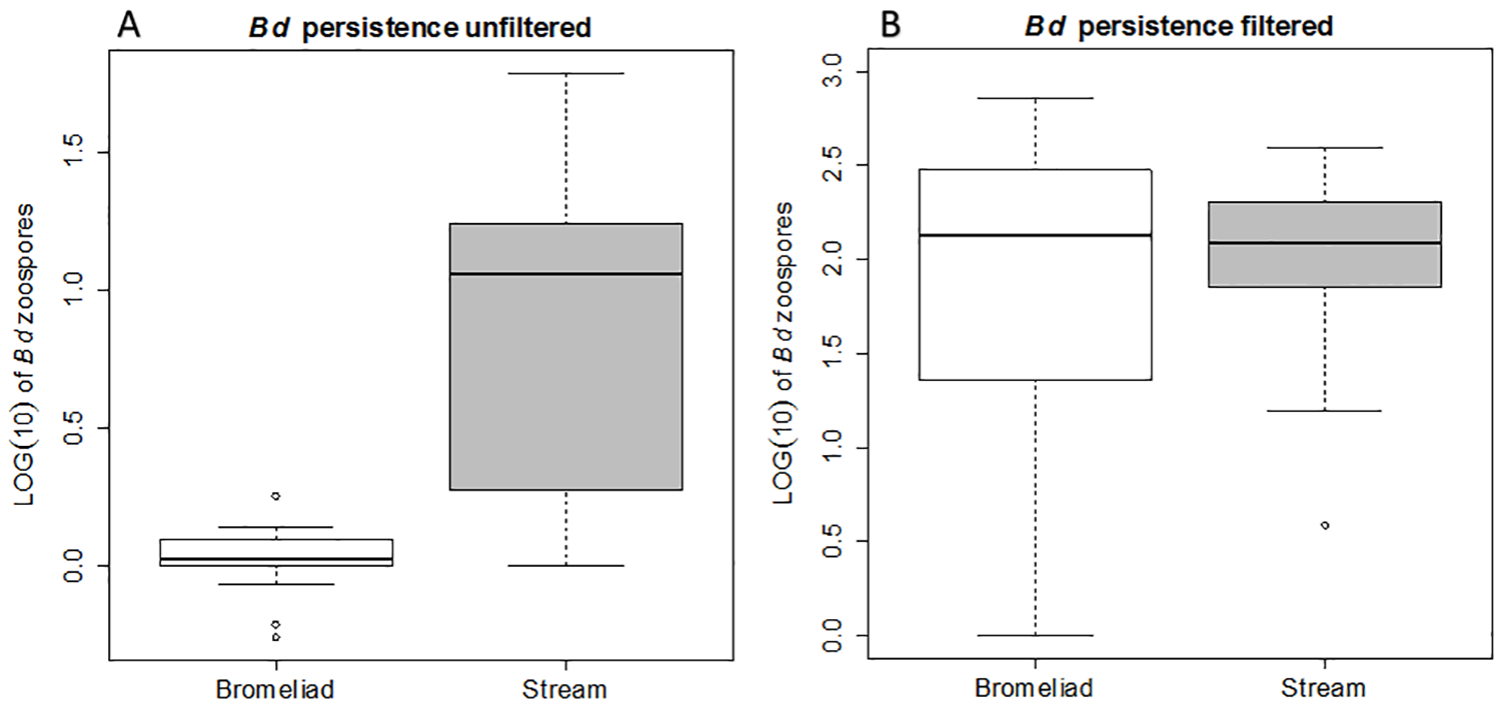
Persistence of *B*. *dendrobatidis* in tank bromeliads versus stream water. For both aquatic microhabitats (bromeliad and stream) the boxplots represent (A) persistence of *B*. *dendrobatidis* in unfiltered water expressed as logarithmic value of genomic equivalents of viable *B*. *dendrobatidis* zoospores, (B) persistence of *B*. *dendrobatidis* in filtered water expressed as logarithmic value of genomic equivalents of viable *B*. *dendrobatidis* zoospores.

Persistence of *Bd* zoospores in water samples from a subset of 15 stream and 15 bromeliad sites after 24 hours was determined by EMA real-time PCR (Fig 3). PCR inhibition did not occur in the EMA samples. Overall, in the stream microhabitat samples, 1.29 log(10) GE (SD of ±0.93 GE) of viable *Bd* zoospores were recovered on average, while 1.97 log(10) GE (SD of ±0.56) of viable *Bd* zoospores were collected on average when the water was first filtered. For the bromeliad water samples, 0.30 log(10) GE (SD of ±0.52 GE) and 1.80 log(10) GE (SD of ±0.96 GE) of *Bd* zoospores were recovered for the unfiltered and filtered conditions respectively. Statistical analyses revealed significant differences between unfiltered and filtered conditions for bromeliads (Mann Whitney Wilcoxon tests, W = 199, Bonferroni adjusted *p* < 0.05) as well as a significant difference between unfiltered conditions of the stream and bromeliad microhabitats (W = 178, Bonferroni adjusted *p* < 0.05). No significant difference was found between unfiltered and filtered conditions for stream (W = 163, Bonferroni adjusted *p* = 0.15) or between filtered conditions of the two microhabitats (W = 112, Bonferroni adjusted *p* = 1).

### Potential abiotic and biotic parameters steering differential survival of *Bd* in bromeliad and stream microhabitats

We surveyed a total of 32 bromeliad sites and 24 stream sites for physicochemical water parameters, and for all of these except one bromeliad site, also data on the micro-eukaryote community diversity were available from microscopic examination. The stream sites were identical to those surveyed for amphibians and *Bd* prevalence (no amphibians found at six of the sites); of the 32 bromeliad sites surveyed, 21 hosted no amphibians (but *Bd* data from amphibians collected in nine additional bromeliad sites were included; see above).

**Fig 4:**
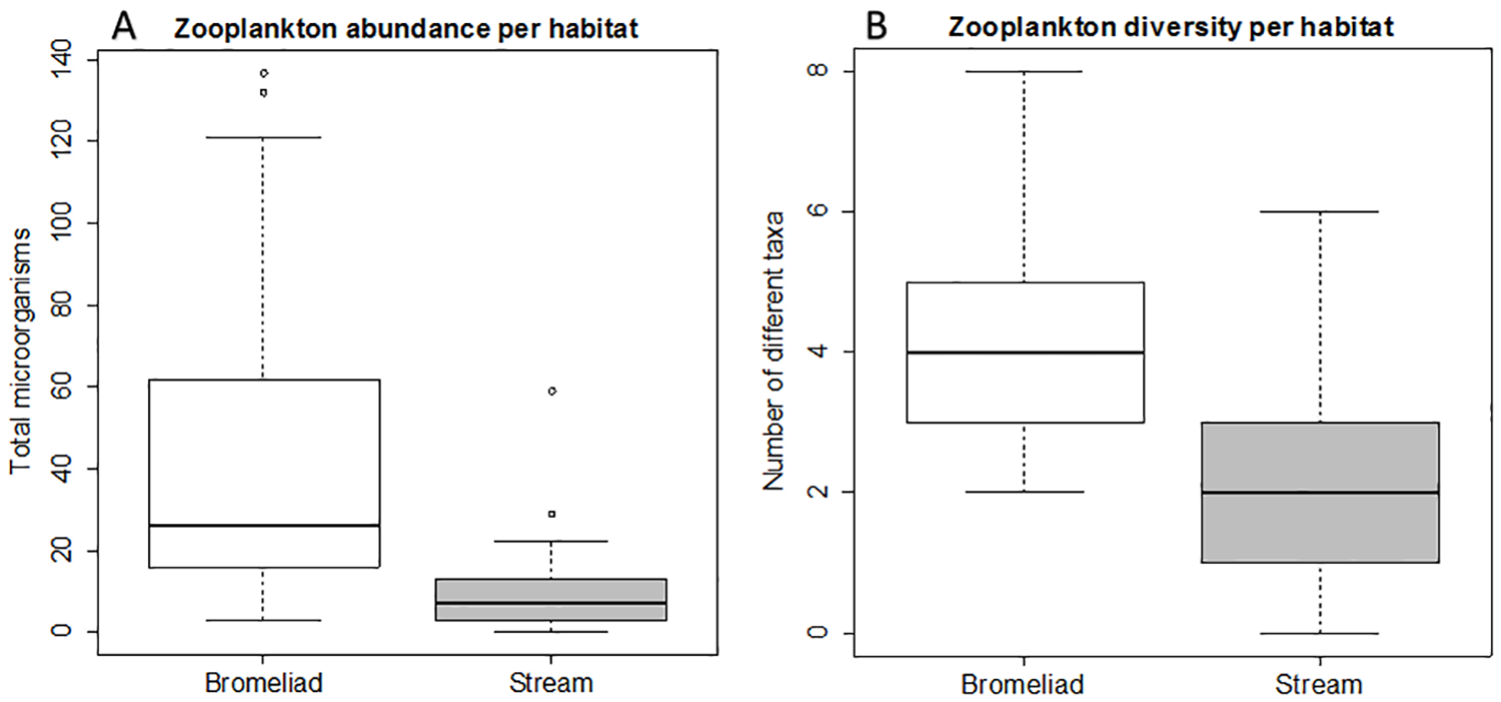
Boxplots showing micro-eukaryote diversity and abundance estimates using microscopy. For both aquatic microhabitats (bromeliad and stream) the boxplots represent (A) micro-eukaryote abundance expressed as micro-eukaryote count per ml (Bromeliad n = 31, Stream n = 24), (B) micro-eukaryote diversity expressed as number of different taxa per ml (Bromeliad n = 31, Stream n = 24).

Temperatures were significantly higher and pH significantly more acidic in bromeliads when compared to stream microhabitats (Mann Whitney Wilcoxon, W = 687.5, *p* < 0.001, W = 741, *p* < 0.001, respectively). All values were within the growth tolerance limits of *Bd*, however, bromeliads had a greater variation in pH and temperature than streams. Canopy openness scores were not significantly different between bromeliad and stream microhabitats (W = 352, *p* = 0.59). Micro-eukaryote abundance and diversity were significantly higher in bromeliad microhabitats in comparison to stream microhabitats with average abundances of 44.2 and 10.6 organisms (SD ± 40.1 and ± 12.6) and average diversities of 4.1 and 2.0 taxa per ml (SD ± 1.4 and ± 1.3) respectively (Fig 4A and 4B; abundance: W = 647, *p* < 0.001, diversity: W = 650, *p* < 0.001).

Micro-eukaryote community diversity estimates using amplicon-based sequencing revealed that protist diversity was higher in stream water samples (1 liter) compared to the bromeliad water samples (30 ml) (all metrics, Kruskal-Wallis test, *p* < 0.001, S1 Fig). Arthropod-rotifer diversity, however, tended to be greater in the bromeliad water samples, even given the small volume examined (Chao1 & OTU Richness, *p* < 0.01; Shannon Diversity, p = 0.92, S1 Fig).

### Micro-eukaryotes present in bromeliad microhabitats limit survival of *Bd* zoospores

**Fig 5:**
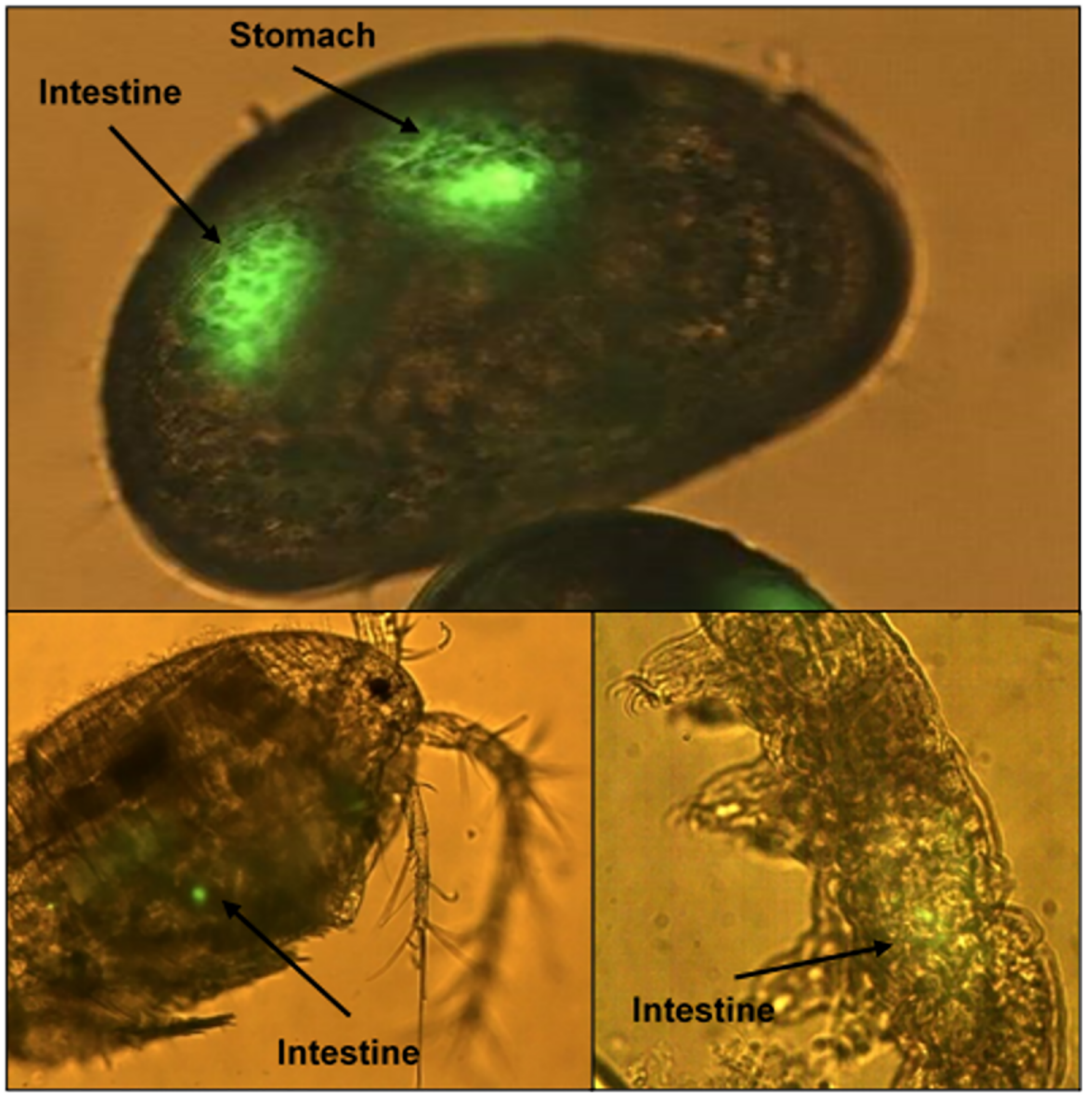
Micro-eukaryotes showing ingestion of fluorescently labelled zoospores of *B*. *dendrobatidis*. Fluorescence microscopy images with arrows indicating presence of fluorescently labelled *B*. *dendrobatidis* zoospores in different sections of the digestive tract. Top: *Cypridopsis* spp., left: *Cyclops* spp., right: *Milnesium tardigradum*. Scale bar represents 100 μm for the upper and left image, and 50 μm for the right image.

To test if the identified micro-eukaryotes are capable of consuming *Bd* zoospores, fluorescently-labelled zoospores were exposed to ostracods, copepods, brown planarias, nematodes and tardigrades. The micro-eukaryote species chosen for this experiment had a high similarity in size and general habits to those encountered in the water samples. Of the tested micro-eukaryotes, ostracods, copepods and tardigrades showed ingestion of fluorescently-labelled *Bd* zoospores (Fig 5).

## Discussion

Within Central America, *Bd* has had devastating effects on amphibian biodiversity [3,9]. However, within this region, records show that stream-associated anuran species are considerably more likely to be categorized as critically endangered compared to those associated with tank bromeliads (S1 Table; OR of 8.88), and also significantly more threatened by *Bd* (S1 Table; OR of 4.79). Although the IUCN dataset used for these calculations also include non-peer reviewed literature and expert opinions, it does indicate a clear difference in the conservation status and susceptibility to *Bd* of bromeliad- and stream-associated amphibians in Central America. This agrees with previous studies suggesting that Central American amphibian species associated with bromeliads may be less affected in *Bd* outbreaks than riparian species in the same region [15–18]. Within Cusuco National Park (CNP), this same trend is observed: there are three declining riparian amphibian species in which chytridiomycosis is a suspected driver, while no negative population trends are reported for the bromeliad-associated species [18,25]. Here, we also show significantly lower prevalence of *Bd* in bromeliad species (3.4%) in comparison to stream-associated species (12.4%). Since only two bromeliad-associated amphibian species exist in CNP at the sampled elevation, and account for prevalence of *Bd* in this microhabitat for this study, differential intrinsic susceptibility of CNP’s amphibian species for *Bd* could be proposed as the underlying cause for the observed differences in *Bd* prevalence. However, taking into account all bromeliad- and stream-associated amphibian species of Central America, the same trend in differential effects of *Bd* on these species is observed, indicating other shared disease-steering factors associated with the aquatic habitat types.

Although several hypotheses on the origins of these differential effects of *Bd* have been proposed, it is likely brought forth by a multitude of infection and disease influencing factors. It has to be noted that our data did not support a significant difference in infection intensity between bromeliad- and stream-associated amphibian species. This could indicate that the differences in disease dynamics of *Bd* between bromeliad- and stream-associated amphibian species are restricted to prevalence only. However, during this study, mostly larval and metamorphic individuals were sampled (Table 1), and divergent infection intensities of *Bd* might be found in adult specimens. Outside of its amphibian host, *Bd* relies on aquatic environments for survival and dispersal [53,5]. While the life stages of *Bd* present in the amphibian skin are rather robust, the motile zoospores released in the environment lack a cell wall and are relatively fragile [54,55]. Therefore, it is plausible to assume that environmental influences are able to exert a significant pressure on the dynamics of chytridiomycosis by affecting environmental *Bd* zoospore loads. Distinct differences in abiotic and biotic variables in stream and bromeliad aquatic microhabitats, could potentially help explain why bromeliad-associated anuran species are able to endure in areas with chytridiomycosis-driven declines, where syntopic riparian species cannot. The tanks present in bromeliads only hold small volumes of water, making these aquatic microhabitats more prone to undergo rapid and frequent fluctuations in physicochemical composition in comparison to relatively stable stream microhabitats [56–58].

In this study, the recorded physicochemical parameters were significantly different between bromeliads and streams, with bromeliads being warmer (18.0°C and 16.3°C, respectively) and more acid environments (4.9 and 6.1, respectively) on average. While no conditions outside of *Bd*’s tolerance limits were recorded, poor growth of *Bd* is described at all recorded pH values of the bromeliad microhabitats, while all pH values recorded for the stream microhabitats fell in the range considered optimal for growth of *Bd* (pH: 6–8) [53], making the bromeliad microhabitats comparatively less suitable for *Bd* survival. Furthermore, even more extreme pH values are known to occur in bromeliad phytotelmata in CNP [58]. The ecological relevance of the pH range considered optimal for *Bd*, as determined under laboratory settings, remains uncertain however [27]. This also applies for temperature. Although the warmer water temperature in bromeliads would suggest that they make more suitable environments for *Bd* proliferation in comparison to stream microhabitats based on *Bd*’s experimentally determined optimal growth temperature range (17–25°C), in nature an increased impact of *Bd* is associated with comparatively lower environmental temperatures [53, 59, 60]. Also, temperature fluctuation, which is expected to occur more frequently and to a greater extent in the small waterbodies in bromeliads, has been determined as a relevant driver of *Bd*’s infection and disease dynamics [24]. Although the overall effects of temperature fluctuation on both host and pathogen are complex and hard to predict [61,62], environments that show broader temperature regimes, and more specifically those that show higher maximum temperatures, are predicted to be less conducive for *Bd* survival [63].

Another environmental parameter shown to affect the infection and disease dynamics of *Bd*, is the abundance of organisms that prey upon/ingest *Bd* zoospores [28,64,65]. In the two distinct sources of water utilized by amphibians in CNP, predatory micro-eukaryotes were more common and diverse in bromeliads (Fig 4A and 4B, S1 Fig). Despite the relatively small sample volume for bromeliads (30 ml in comparison to 1 liter for stream samples) higher diversities of micro-arthropods and rotifers were found in comparison to stream samples with 18S rRNA diversity estimates. Protist diversity estimates were significantly higher in stream samples. While certain protists are known to be able to effectively reduce environmental *Bd* zoospore loads (for example *Paramecium Aurelia* [28]), a large portion of the protist taxa likely have no effect on *Bd* zoospores due to their feeding strategy or autotrophic life style. Furthermore, relatively high diversity estimates with the 18S method in comparison to the microscopic method, might occur due to detection of DNA of organisms not visually present at the time of microscopic examination. The diversity estimates correlate with significant differences in the prevalence of *Bd* in amphibians encountered in the two microhabitats (Table 1) and with differential persistence of *Bd*, with bromeliads acting as a significantly more hostile environment (Fig 3). When predatory micro-eukaryotes were removed from the water by filtration, survival of *Bd* in tank bromeliad water and stream water were equal (Fig 3). Ingestion of *Bd* zoospores by commercially available micro-eukaryotes that resembled species found in the studied microhabitats further strengthens our hypothesis that a multitude of organisms belonging to a vast diversity of taxa are able to reduce environmental loads of *Bd*.

In this study, we have used univariate comparisons of response variables (*Bd* prevalence and infection intensity) and possible predictor variables (temperature, pH, micro-eukaryote communities) among bromeliad and stream sites. While multivariate regression-based models would a priori be a more powerful approach to test for causal relationships among these variables, such analyses are prevented by the structure of the data in our study, i.e., by the absence of amphibians in many bromeliad sites, the presence of single amphibian individuals in others, and the lack of biotic and abiotic data for additional bromeliad sites from which amphibians were sampled. Furthermore, because different species of amphibians were sampled from bromeliad and stream sites, effects due to common ancestry or host effects on *Bd* infection prevalence cannot be fully excluded either. Differences in risk between bromeliad and stream amphibians could be biased due to more closely related species sharing increased susceptibility to *Bd* [66]. Although the relations between *Bd* infection, habitat type, and biotic and abiotic habitat parameters thus require further testing in this system, our experimental results suggest that the aquatic microhabitats present in bromeliads in CNP are less conducive for *Bd* survival. We therefore hypothesize that the combined biotic and abiotic differences among habitat types are likely steering local *Bd* infection and disease dynamics in CNP. By providing environments in which the build-up of a high infection pressure is inhibited, the steep increase in *Bd* prevalence and infection intensity as witnessed in classical chytridiomycosis outbreak scenarios [2,9,10] may be prevented, and as such, specialized microhabitats like bromeliad phytotelmata could therefore contribute in creating chytridiomycosis-safe refuges in which amphibian species (especially specific microhabitat specialists) can endure during surrounding chytridiomycosis epidemics. This theory thus adds to existing hypotheses such as, differences in species susceptibility to *Bd* and variable disease dynamics due to differences in host densities, to help explain *Bd* infection and disease dynamics [20,67, 68].

Combined with the study of Schmeller et al. [28], the fact that micro-eukaryotes contribute to creating an aquatic environment not conducive to *Bd* zoospore survival in two dramatically different scenarios of chytridiomycosis-driven declines (Pyrenees vs Central American cloud forest), suggests that this may be a common determinant of *Bd* infection dynamics. This knowledge may be applied in chytridiomycosis mitigation efforts by augmentation of natural aquatic habitats to reduce the effect and/or severity of chytridiomycosis where applicable. Although environmental augmentation of existing aquatic habitats is undesirable for the majority of areas most severely impacted by chytridiomycosis, and likely difficult and/or impractical for certain types of water bodies used by amphibians, creating or supplying *Bd* safe zones in problem areas may be worth exploring. This study illustrates the importance of increasing our understanding of the complex interactions at play in the infection and disease dynamics of *Bd* in natural amphibian communities and habitats, and could potentially prove to be valuable for the development of chytridiomycosis mitigation strategies by managing aquatic habitats.

## Supporting information

S1 Fig Boxplots showing 18S rRNA microeukaryote diversity and richness estimates for bromeliad and stream microhabitats for rarefied datasets (1000 reads per sample). Plots show total number of operational taxonomic units (OTUs), Shannon diversity, and Chao1 diversity per sample. Number of OTUs represents an uncorrected representation of the number of different microeucaryotes per sample (i.e., species richness), whereas the Chao1 index estimates richness, i.e., the total number of species present in a community, by correcting species richness based on the number of singletons (an OTU represented by a single read in a sample), assuming that such singletons indicate the species inventory is incomplete. The Shannon index is a community diversity index combining species richness and abundance into a single value of evenness.
(DOCX)

S1 Table Bromeliad- and stream-associated Central American anuran species included in the assessments of IUCN conservation status and of occurring threats, with their corresponding IUCN categories, larval development or adult association to the habitat and threats. (XLSX)

S2 Table Data collected from the bromeliad and stream sampling locations. Data includes physicochemical parameters, Bd prevalence and infection intensity and micro-eukaryote abundance and diversity.
(XLSX)
