# Supplementary material for: Host niche may determine disease-driven extinction risk
Source: PLoS One. 2017 Jul 13;12(7):e0181051. doi: 10.1371/journal.pone.0181051 (PMC5509289; doi:10.1371/journal.pone.0181051)
Supplement: S1 Fig — Plots show total number of operational taxonomic units (OTUs), Shannon diversity, and Chao1 diversity per sample. Number of OTUs represents an uncorrected representation of the number of different microeucaryotes per sample (i.e., species richness), whereas the Chao1 index estimates richness, i.e., the total number of species present in a community, by correcting species richness based on the number of singletons (an OTU represented by a single read in a sample), assuming that such singletons indicate the species inventory is incomplete. The Shannon index is a community diversity index combining species richness and abundance into a single value of evenness. (DOCX) [file pone.0181051.s001.docx]

**Figure S1: Boxplots showing 18S rRNA zooplankton diversity estimates for bromeliad and stream microhabitats.**
